# Two single group, prospective, baseline-controlled feeding studies in infants and children with chronic diarrhea fed a hypoallergenic free amino acid-based formula

**DOI:** 10.1186/1471-2431-14-136

**Published:** 2014-05-29

**Authors:** Marlene W Borschel, Dean L Antonson, Nancy D Murray, Maria Oliva-Hemker, Lynn E Mattis, Benny Kerzner, Vasundhara Tolia, Geraldine Baggs

**Affiliations:** 1 Scientific & Medical Affairs, Abbott Nutrition, Abbott Laboratories, 3300 Stelzer Road, Columbus, Ohio 43219, USA; 2 Pediatric Gastroenterology of Omaha, University of Nebraska Medical Center, Omaha, NE, USA; 3 Neonatal Care, PC, 717 N 19th Plaza, Omaha, NE, USA; 4 Division of Pediatric Gastroenterology and Nutrition, Johns Hopkins University School of Medicine, Baltimore, MD, USA; 5 Division of Pediatric Gastroenterology and Nutrition, Johns Hopkins Hospital, Baltimore, MD, USA; 6 Department of Pediatric Gastroenterology, Children’s National Medical Center, Washington, DC, USA; 7 Providence Hospital, Southfield, MI, USA

**Keywords:** Free amino acid-based formula, Hypoallergenic, Elemental formula, Chronic diarrhea, Short bowel syndrome, Malabsorption, Eosinophilic gastroenteritis, Food allergy, Inflammatory bowel disease, Gastroesophageal reflux

## Abstract

**Background:**

Infants and children with chronic diarrhea (CD) often require specialized foods or parenteral nutrition (PN) to achieve adequate nutrient intakes to support growth and development. We assessed the efficacy of an amino acid-based formula (AAF) in supporting growth and improving symptoms in infants and children with CD from multiple etiologies.

**Methods:**

Two studies were conducted: CD study in children (CD-C) and CD study in infants (CD-I). Each was a single group, baseline-controlled study in which each subject served as his/her own control. At enrollment, all subjects had CD lasting > 2 weeks and had ≥ 4 stools/day. Subjects were fed an AAF for 80 days starting at SD5, and were assessed at SD 28 and 84.

**Results:**

CD-C: 18 of 19 subjects completed the study. At enrollment, the mean age was 5.6 ± 0.7 years, the most common diagnosis was short bowel syndrome (SBS) (n = 13), and 5 subjects with SBS were on PN. Subjects achieved significant increases in weight-for-age z-scores (p = 0.026). Over 50% of subjects achieved improvements in clinical outcomes targeted most frequently by their physicians. Of the five subjects on PN at enrollment, four had substantial weight gain and four had their PN requirements decreased. CD-I: 22 of 27 subjects completed the study. At enrollment, the mean age was 3.3 ± 0.3 months, the most common diagnosis was food allergy (n = 20), and no subjects were on PN. Subjects achieved significant increases in weight-for-age z-scores (p = 0.0023), significant decreases in the number of stools/day (p = 0.0012), and improvements in stool consistency (p = 0.0024). Over 80% of subjects achieved improvements in the clinical outcomes targeted most frequently by their physicians.

**Conclusions:**

Infants and children with CD fed an AAF for three months displayed significant improvements in weight-for-age z-scores and clinical symptoms. Children dependent on PN also grew well and four of five decreased their dependence on PN.

**Trial registration:**

Both trials were registered on ClinTrials.gov (CD-C, NCT01812629; CD-I, NCT01820494).

## Background

The terms chronic diarrhea (CD), persistent diarrhea, and toddler’s diarrhea are often used interchangeably to describe various bowel disorders with multiple etiologies. The generally accepted definition is diarrhea lasting at least two weeks [1-4]. The causes of CD can be divided into four principal pathophysiologic mechanisms: osmotic, secretory, dysmotility-associated and inflammatory [4]. There is little data to indicate that the incidence of CD is decreasing, however, Bhutta et al. [1] noted that there has been a significant reduction in the number of publications on the topic.

Infants and children with CD often require specialized medical foods to achieve adequate nutrient intakes to support growth and development. Parenteral nutrition (PN) may be used [2], but to minimize the risk of complications related to dependence on PN, increasing volumes of enteral nutrition must be introduced and carefully managed. Amino acid-based formulas (AAF) can alleviate diarrhea due to food allergies [5,6], although, there is a paucity of evidence regarding the use of these formulas to treat pediatric patients with other forms of CD. The objective of these studies was to assess the efficacy of a specific AAF in supporting growth and improving symptoms in infants and children with CD from multiple etiologies.

## Methods

Two individual studies were conducted in subjects with CD: Chronic Diarrhea Study in Children (CD-C) and Chronic Diarrhea Study in Infants (CD-I). Each study was a single group, prospective baseline-controlled feeding study in which each subject served as his/her own control. Study visits were at study days (SD) 1, 28 and 84. All subjects received a clinically-labeled AAF that was fed for 80 days following SD5. The primary objective of the studies was to assess the growth of subjects who received the study feeding as the primary feeding for three months. Other assessments included formula intake, tolerance of study feeding, stooling patterns and consistency, physician evaluation of clinical symptoms, and blood biochemistry levels.

### Subjects

Subjects were eligible for enrollment if they met the following criteria at enrollment: ≤12 months of age (CD-I), ≥12 months of age (CD-C), had chronic diarrhea lasting >2 weeks and ≥ 4 stools/day; had been diagnosed with at least one of the following conditions where diarrhea is common and AAF may be useful in the management of some patients with the condition: short-bowel syndrome (SBS), eosinophilic gastroenteritis, food allergy, inflammatory bowel disease, pancreatic disease, protein maldigestion, HIV-advanced disease, or other gastrointestinal disease requiring an elemental diet. Parents agreed to give the study feeding as the primary source of calories during the study. There was no restriction regarding the type of feeding prior to enrollment. Feedings were given *ad libitum*. Administration of medications that would prevent accurate measurements of tolerance and/or growth (e.g., corticosteroids) was not permitted.

For CD-C subjects: Diets were individualized based on clinical condition and nutritional needs. All subjects were considered to be clinically stable. Supplemental PN, solid foods and beverages were permitted. Additional enteral modular supplements could be added to the study formula if the total calories were increased by no more than 15%. The study feeding was to provide at least 50% of energy during the study. Vitamin and mineral supplements were allowed and their use recorded. The feeding dilution was modified to meet each subject’s individual needs. The study feeding was used to replace the primary enteral feeding used at baseline and there was no intent to significantly change the current feeding regimen.

For CD-I subjects: No other complete enteral feedings or human milk were allowed. Additives to the study feeding such as medium-chain triglycerides were allowed but were not to increase the caloric density of the study feeding by >15%. Solid foods were allowed. Records were kept and analyzed to ensure that subjects received at least 50% of total energy from the study feeding.

Parents or guardians provided written informed consent prior to enrollment. Subjects for the CD-C study were recruited and followed as outpatients at two tertiary care pediatric gastroenterology clinics in Baltimore, Maryland and Washington, DC, and the study protocol was approved by the Institutional Review Boards at Johns Hopkins Hospital and The Children’s Research Institute at Children’s National Medical Center. Subjects for the CD-I study were recruited from pediatric gastroenterology clinics in Baltimore, Maryland; Omaha, Nebraska; and Detroit, Michigan, and the study protocol was approved by the Institutional Review Boards at Johns Hopkins Hospital, University of Nebraska Medical Center and the Children’s Hospital of Michigan.

### Study feeding

The study feeding was EleCare® Amino Acid-Based Medical Food (Abbott Nutrition, Abbott Laboratories Columbus, Ohio) provided in powdered form. The cans and cases were clinically-labeled and identified only by clinical product number. The investigators were not blinded to the formula used in the study. The macronutrient sources and nutrient content of EleCare are shown in Table 1.

**Table 1:** **Nutritional composition of study formula at 20 and 30 kcal/fl oz**^**a**^

**Macronutrient**	**Source**	**Content at 20 kcal/fl oz (g/L)**	**Content at 30 kcal/fl oz (g/L)**
Protein equivalent	100% free L-amino acids	20.3	30.1
Carbohydrate	100% corn syrup solids	72.3	107.0
Fat	38% high-oleic safflower oil, 33% medium chain triglycerides, 29% soy oil	32.2	47.6

^a^Values represent label claim. Standard dilution for infants is 20 kcal/fl oz and 30 kcal/fl for children over 1 year of age. Information is given for example only as feeding dilutions and mixtures were individualized to meet each subject’s needs.

### Study assessments

After enrollment on SD1, parents were instructed to continue feeding the subject’s current feeding from SD1 through SD4, during which baseline information was collected. Study feedings were initiated on SD5, and subsequent visits were at SD28 (±3 days) and SD84 (±3 days). At each visit, parents were given enough EleCare to last until the next visit. At SD1, 28 and 84, a physician assessment was performed, and subjects were weighed and length was measured by trained personnel. Children who could stand without assistance were weighed twice to the nearest 0.1 kg on a calibrated scale wearing light undergarments. Height was measured twice to the nearest 0.1 cm in the standing position using a measuring board attached to a vertical surface. Infants were weighed twice in the nude to the nearest 1.0 g on a calibrated electronic scale. Infant length and length of young children unable to stand for the height assessment were measured twice to the nearest 0.1 cm with the child in a recumbent position using a pediatric length board. A third measurement was taken if weights differed more than 0.01 kg or lengths differed more than 0.4 cm. World Health Organization (WHO) z-scores were determined. Three-day dietary and stool records were completed for SD1-4 (baseline), SD5-7, SD25-27 and SD81-84. These records included the number of stools per day, and characteristics (consistency and frequency) of the stools, volume of study feeding consumed at each feeding, consumption of foods other than the study feeding, and incidences of regurgitation (CD-C), spit-up (CD-I), and vomiting. For subjects with ostomies, parents recorded the volume of stool collected instead of stool number, and they did not record stool consistency. Mean rank stool consistency (MRSC) was calculated from the parental ranking of each stool using a scale of 1 to 5 for CD-C (1 = runny, mostly liquid, 2 = soft, unformed, 3 = soft, formed, 4 = hard, formed, 5 = hard, dry) and a scale of 1 to 4 for CD-I (1 = watery, 2 = loose/mushy, 3 = soft, 4 = formed). Stool records provided descriptions of each stool category to assist parents in accurate recording. The MRSC was calculated for each day, and the average of these daily means was calculated over the study period.

At the SD1 visit, a physician assessed each subject and determined the clinical outcome improvements that were to be targeted for each subject. At the SD84 assessment, physicians determined if the subject had met the targeted goal(s) or improved in other significant ways during the study.

For CD-I subjects, an interval history was conducted by telephone on approximately SD56 to determine if any adverse events had occurred since the last visit, and if there were any changes in medications or nutritional intake since the last visit.

### Hematological measurements

A blood sample was collected by venipuncture on SD1, 28 and 84 (CD-C) or on SD1 and 84 (CD-I). Measurements included hemoglobin, hematocrit, and serum levels of albumin, total protein, urea nitrogen and alkaline phosphatase for CD-C and CD-I, and serum uric acid, and white blood cell and platelet count for CD-C. Samples were analyzed by each site’s hospital laboratory using standard methodologies.

### Statistical analyses

CD-C: Each subject served as his/her own control, thus the analyses were conducted on subjects who completed the study and had SD1 and SD84 measurements. Changes in continuous variables from SD1 to SD84 were analyzed using Student’s paired t-test or Wilcoxon’s signed rank test for highly skewed distributions. Categorical/ordinal data were analyzed using Friedman’s chi-square, and weight and length were also analyzed using a random coefficients model with SAS PROC MIXED. Changes in blood chemistry status levels (within or outside of reference ranges) between SD1 and SD84 were analyzed using McNemar’s test. Statistical comparisons were considered significant at the 0.05 probability level; no adjustments were made for multiplicity of testing. Data are reported as the mean ± SEM unless otherwise indicated.

## Results

### CD-C

Nineteen subjects were enrolled and 18 completed through SD84. The one subject who did not complete the study was dropped due to acute viral enteritis. The mean age of subjects on SD1 was 5.6 ± 0.7 years (range 1.2 to 9.6), and 63% were born at term gestation (Table 2). Some subjects had multiple diagnoses, with the most common diagnoses being SBS (n = 13; all subjects born preterm had SBS), and maldigestion/malabsorption (n = 16). All children with SBS suffered from severe chronic malabsorption (4–20 stools/day) and all had required PN support for a minimum of 14 months at one time while in the care of the investigators. At study entry, eight subjects were consuming a casein hydrolysate-based formula, six were on a different AAF, four were on a complete pediatric liquid nutritional formula, and one was on a complete adult nutritional product. Subjects had been receiving their previous feeding for a mean of 3.1 ± 0.6 years (range 0.1 - 9.6 years) prior to enrollment. Sixteen of the 18 subjects who completed the study consumed the study feeding via tube-feeding.

**Table 2:** Demographic characteristics for subjects enrolled in the study in children (CD-C) and in infants (CD-I)

**Variable**	**CD-C**	**CD-I**
	**N = 19**	**N = 27**
Age at SD1	5.6 ± 0.7 years^a^	3.3 ± 0.3 months
Term gestation, n (%)	12 (63)	20 (74)
Males, n (%)	10 (53)	14 (52)
Ethnicity, n (%)		
Caucasian	10 (53)	24 (89)
African American	8 (42)	2 (7)
Other	1 (5)	1 (4)
Diagnoses^b^		
Short bowel syndrome	13	2
Eosinophilic gastroenteritis	5	7
Food allergy	5	20
Inflammatory bowel disease	2	0
Maldigestion/malabsorption	16	1
Malnutrition	3	2
Gastroesophageal reflux	1	0
Viral enteritis/Post-infectious gut mucosal injury	0	2
Formula Intolerance	0	2

^a^Mean ± SEM.

^b^Some subjects had multiple diagnoses.

For the 18 subjects who completed the study, there were significant improvements in WHO weight-for-age z-scores (p = 0.0260) (Table 3). Mean weight gain increased from 16 ± 10 g/day from SD1-SD28 to 18 ± 6 g/day from SD28-84 (p = 0.7987). Past weight gain was available for 15 of 19 subjects for an average of 90 ± 5 days prior to study entry. Mean weight gain was 7 ± 2 g/day prior to study entry compared with gains of 19 ± 7 g/day during the study period (p = 0.0545).

**Table 3:** Anthropometric, stooling characteristics, intakes and abdominal findings in subjects who completed the study

	**CD-C, n = 18**			**CD-I, n = 22**		
	**Initial**^**a**^	**SD28**	**SD84**	**Initial**^**a**^	**SD28**	**SD84**
Weight, kg	17.8 ± 1.3	18.3 ± 1.4	19.2 ± 1.5^b^	5.73 ± 0.35	6.40 ± 0.33	7.53 ± 0.27^b^
WHO weight-for-age z-score	−0.81 ± 0.20	−0.73 ± 0.22	−0.50 ± 0.23^c^	−0.83 ± 0.29	−0.64 ± 0.26	−0.24 ± 0.20^b^
Length, cm	103.1 ± 4.3	103.5 ± 4.1	104.9 ± 4.2^b^	60.4 ± 1.4	62.8 ± 1.3	66.9 ± 1.0^b^
WHO length-for-age z-score	−1.68 ± 0.40	−1.69 ± 0.36	−1.64 ± 0.38	−0.47 ± 0.31	−0.34 ± 0.29	−0.09 ± 0.24
Number of stools/day^d^	5.5 ± 1.0	5.2 ± 0.8	4.9 ± 1.0	3.5 ± 0.5	2.3 ± 0.3	1.5 ± 0.2^b^
MRSC^e^	1.5 ± 0.1	1.6 ± 0.2	1.7 ± 0.2	2.1 ± 0.1	2.3 ± 0.2	2.7 ± 0.2^b^
Study feeding intake, mL/kg/day	125 ± 14	123 ± 12	121 ± 13	165 ± 9	142 ± 9	130 ± 7^f^
Energy intake from study feeding, kcal/kg/day	101 ± 11	104 ± 12	101 ± 12	105 ± 6	95 ± 6	87 ± 5^f^
Total energy intake, kcal/kg/day	115 ± 12	116 ± 13	110 ± 12	110 ± 5	103 ± 5	104 ± 5
Physicians Evaluation of Abdominal Findings, number (%)^g^						
Subjective Complaints						
None - absent	5 (28%)	14 (76%)	15 (83%)	--	--	--
Mild - complaints of nausea or abdominal pain, no change in activity	8 (44%)	4 (24%)	3 (17%)	--	--	--
Moderate - frequent complaints of nausea or pain, decreased activity	5 (28%)	0	0	--	--	--
Severe - patient in bed, crying or notably distressed	0	0	0	--	--	--
Objective Complaints^h^						
None - absent	0	1 (6%)	1 (6%)	5 (23%)	17 (77%)	22 (100%)
Mild - 1 episode of emesis or 1 more diarrhea stool/day than usual	0	3 (18%)	2 (11%)	1 (5%)	1 (5%)	0
Moderate – 2 to 3 episodes of emesis or 2–3 more diarrheal stools/day than usual or 1 of each/day	3 (17%)	4 (24%)	6 (33%)	5 (23%)	3 (14%)	0
Severe - > 3 episodes of emesis or > 3 diarrheal stools/day than usual or 2 of each/day	15 (83%)	10 (53%)	9 (50%)	11 (50%)	1 (5%)	0

^a^Initial period was SD1 for anthropometric measurements and abdominal findings, and was the baseline period (SD1-4) for stooling patterns and intakes.

^b^Significant improvement during the study, p < 0.01.

^c^Significant improvement during the study, p < 0.05.

^d^Data for CD-C subjects does not include 3 subjects with ostomies.

^e^Mean rank stool consistency based in a 5-point scale for children (CD-C) (1 = runny, mostly liquid, 2 = soft, unformed, 3 = soft, formed, 4 = hard, formed, 5 = hard, dry) and a 4-point scale for infants (CD-I) (1 = watery, 2 = loose/mushy, 3 = soft, 4 = formed).

^f^Significant decrease during the study, p < 0.01.

^g^Significant improvement for CD-C (p = 0.001) and CD-I (p = 0.0002).

^h^Significant improvement for CD-C (p = 0.005) and CD-I (p < 0.0001).

Three subjects had ostomies, and their mean stool volume decreased from 6735 ± 3764 mL/day at baseline to 4508 ± 2552 mL/day at SD84 (p = 0.2194). For the remaining 15 subjects without ostomies, there were non-significant improvements in stooling patterns. From baseline to SD84, the mean number of stools per day decreased from 5.5 ± 1.0 to 4.9 ± 1.0, MRSC increased from 1.5 ± 0.1 to 1.7 ± 0.2 (Table 3), and the percent of stools that were runny, mostly liquid decreased from 57 ± 9% to 49 ± 11%. From baseline to SD84 there were no significant changes in the daily volume of study feeding consumed, energy intake from the study feeding, or total energy intake (Table 3).

From SD1 to SD84 there were significant shifts from more to less severe abdominal complaints (Table 3). Over 50% of subjects achieved improvements in the clinical outcomes targeted most frequently by physicians (Table 4). There were non-significant decreases in the percent of days that subjects experienced regurgitation (22 ± 8% to 13 ± 6% from baseline to SD84), and the percent of subjects regurgitating on any day (39% to 28%). There were no statistically significant changes in blood biochemistry values, or in the percent of subjects whose values were within or outside normal limits during the study.

**Table 4:** Physician-targeted clinical outcomes

**Clinical Outcome**	**CD-C**	**CD-I**
	**n = 18**	**n = 22**
Decreased stool number	16 (69%)	17 (100%)
Decreased vomiting	7 (57%)	6 (83%)
Decreased gas	5 (80%)	4 (100%)
Weight gain	18 (83%)	12 (92%)
Increased calorie intake	4 (75%)	2 (100%)

Data expressed as: number of subjects at baseline having the outcome as target (% of targeted subjects who achieved the targeted outcome).

### Subset of subjects on PN

At entry, five subjects with SBS remained dependent on PN support in order to meet their overall nutritional and hydration needs. Prior to study entry, three of the subjects had consumed a different AAF, one had consumed an extensively hydrolyzed formula, and one had consumed a partially hydrolyzed formula. Four of five had substantial weight gain during the study. Four had their PN requirements decreased; three of these four tolerated an increase in enteral intake during the study. Their mean weight gain was 40.2 g/d during the study, compared with 10 g/d in the three months prior to study enrollment. The percentage of energy these five subjects received from PN decreased by 50%, 50%, 26% and 17% and 0%, respectively, during the study, while energy from enteral nutrition increased. Mean calories from PN decreased from 50 kcals/kg/day at SD1 to 32 kcals/kg/day at SD 84. Mean calories from enteral nutrition were 95 kcals/kg/day at SD1 and 92 kcals/kg/day at SD84.

### CD-I

Twenty-seven subjects were enrolled and 22 completed through SD84. Two subjects never consumed the study feeding, one consumed less than 50% of total caloric intake from the study feeding, and two were removed from the study early by their parents for reasons not related to the study feeding. The mean age of enrolled subjects on SD1 was 3.3 ± 0.3 months (range 0.7 to 8.4), 74% were born at term gestation (Table 2), and the mean birth weight was 3184 ± 142 g. The most common diagnoses were food allergy (n = 20) and eosinophilic gastroenteritis (n = 7). On SD1, 12 subjects were on a different AAF, 13 were on a casein hydrolysate, one was on a combination of a different AAF and casein hydrolysate, and one was on a partial whey hydrolysate formula. On SD1, one subject had an ostomy, four were tube-fed, and none were receiving PN; one subject was receiving PN on SD28 but not at SD84 because of an elective colostomy for management of Hirschsprung’s disease.

From SD1 to SD84 WHO weight-for-age z-scores improved significantly, increasing from -0.83 ± 0.29 to -0.24 ± 0.20 (p = 0.0023) (Table 3). Mean weight and length gains were 21.4 ± 1.8 g/day and 0.77 ± 0.07 mm/day, respectively, during the study. From baseline to SD84, the mean number of stools per day decreased significantly from 3.5 ± 0.5 to 1.5 ± 0.2 /day (p = 0.0012), MRSC increased significantly from 2.1 ± 0.1 to 2.7 ± 0.2 p = 0.0024), and total daily energy intake was maintained from 110 ± 5 to 104 ± 5 kcal/kg/day at baseline and at SD84, respectively (Table 3). There were significant decreases (p < 0.01) in the volume of study feeding and energy intake from study feeding from baseline to SD84 (Table 3).

From SD1 to SD84 there were significant shifts from more to less severe objective abdominal complaints (Table 3). For the physician-targeted outcomes, 100% of subjects achieved decreases in stool number and gas, 100% achieved increased calorie intake and over 80% achieved weight gain and decreased vomiting (Table 4). All blood analytes were within references ranges at each time point, with serum alkaline phosphatase levels decreasing from SD1-84, and the remaining analytes increasing (p > 0.05; data not shown).

## Discussion

In this prospective, three month feeding study, growth was significantly improved in subjects with CD fed EleCare. The children had significant improvements in WHO weight-for-age z-scores, while the infants had significant improvements in weight-for-age z*-s*cores, number of daily stools, and stool consistency. There were significant shifts from more to less severe abdominal complaints for both groups. These findings extend those of Borschel et al. [5] reporting growth of a large cohort of infants exclusively fed EleCare during the first 4 months of life and those from Sicherer et al. [6] reporting that EleCare maintained growth in 18 children with eosinophilic gastroenteritis and/or multiple food allergy. Our findings also demonstrate the efficacy of EleCare in improving symptoms in pediatric subjects with CD.

AAFs are used to treat a variety of conditions, including diarrhea from food allergies. Over 90% of the infants in our study had food allergy. Numerous studies have reported that AAF supports growth and alleviates diarrhea due to food allergies [7,8], and they are preferable to extensively hydrolyzed formulas for certain patients [7-10]. An additional incentive to use AAF is that allergic responses can occur with extensively hydrolyzed formulas because they may contain residual peptides that can elicit an allergic response in sensitized children [11-14]. Sicherer et al. [6] has reported that EleCare was safe for the management of 31 children with cow’s milk and multiple food allergy. We have previously reported a significant decrease (p < 0.0001) in the daily number of stools in infants with food protein-induced eosinophilic proctocolitis who were switched to EleCare [15]. The present study provides additional support for the efficacy of EleCare in managing infants with food allergy.

Whereas the use of AAF in food allergy is well documented, there is a paucity of data related to their efficacy in treating CD due to other causes. The recent report by Singh et al. [16] that microscopic colitis is present in some children with CD suggests that AAF may also be efficacious in alleviating CD in children with this pathologic condition. The study cohort in the CD-C trial was unique in several ways. Most of the children enrolled had complex gastrointestinal disease with severe fluid and nutrient malabsorption. Sixty-eight percent of subjects had SBS and 38% of these required some PN, thus, malabsorption was much more severe than what would typically be seen in children with other conditions causing CD. The majority of children required a readily absorbed AAF or semi-elemental formula due to significantly reduced intestinal absorptive surface area, intestinal inflammation, or both, as well as continuous infusion of tube feedings round-the-clock to maximize absorption and meet their nutritional and high caloric requirements. Most children also had a history of formula intolerance including vomiting, abdominal distention and discomfort, and severe watery diarrhea. Several subjects suffered from intestinal dysmotility due to previous ischemic injury to the residual bowel, which affected enteral feeding tolerance. All were relatively stable at home and most were followed closely by a Home Nutrition Support Team. At study entry, several of the subjects had reached a threshold in terms of the amount of enteral feedings which could be tolerated, which precluded ongoing enteral feeding advancement with their baseline formula. As the subjects were clinically stable at the time of enrollment, it was unexpected that most of the children experienced increased weight gain simply as the result of a change in enteral formula. The significant improvements in weight achieved by this study population may be due to a combination of factors: 1) improved efficiency of nutrient absorption with the combination of free amino acids, medium-chain- and long-chain-triglycerides in EleCare, 2) decreased allergic inflammation in a susceptible population with subsequent enhanced absorption, and 3) increased caloric retention or increased caloric intake secondary to decreased incidence of vomiting and other feeding intolerance.

Five of the children in the CD-C study were on PN at enrollment, each of whom had SBS. SBS is the most common cause of intestinal failure in children, and the complex medical management of these patients includes advanced nutritional support [17]. PN is often used to provide nutrients for growth and development while the bowel undergoes adaptation necessary to transition to an enteral diet. However, PN is associated with significant morbidity, mortality and financial costs [18]. The duration of dependence on PN is significantly related to the percent of energy intake received enterally [19], however, transitioning children with SBS from PN to enteral nutrition remains a difficult challenge. In the current study, impressive improvements in growth were seen in the children with SBS who were dependent upon PN at study entry. Their mean weight gain increased more than four-fold compared with the three months prior to study enrollment, and they were able to substantially decrease calories from PN without comparable increases in enteral energy intake.

There is a paucity of literature regarding successful strategies for weaning patients with SBS from PN to enteral nutrition. However, our finding that an AAF facilitated this process is in agreement with those of others. Andorsky et al. [20]. reported that enteral feeding with breast milk or an AAF was associated with shorter duration of PN for infants with SBS, while Bines et al. [21] reported that four children with SBS who had persistent feeding intolerance to an extensively hydrolyzed formula and who were changed to an AAF were able to cease PN within 15 months, stool output decreased dramatically, enteral contribution to energy intake increased, and patient morbidity and hospitalization decreased.

A limitation of our study is the small samples sizes and the heterogeneity of subjects’ clinical conditions. However, this heterogeneity can also be a strength, demonstrating the efficacy of EleCare for a broad range of clinical indications or varying severity. This was particularly evident, in the children in the CD-C trial.

## Conclusions

In summary, we report that children and infants with CD fed EleCare for three months displayed improvements in growth and clinical symptoms. A notable finding is that stable PN-dependent children with SBS grew well and decreased their dependence on PN during the study. Additional clinical trials enrolling greater numbers of subjects are needed to determine if longer-term clinical benefits of AAF can be achieved.

## Abbreviations

AAF: Amino Acid-Based Formula; CD: Chronic Diarrhea; CD-C: Chronic Diarrhea Study in Children; CD-I: Chronic Diarrhea Study in Infants; MRSC: Mean Rank Stool Consistency; PN: Parenteral Nutrition; SBS: Short Bowel Syndrome; SD: Study Day; WHO: World Health Organization.

## Competing interests

MWB and GEB are employees of Abbott Nutrition, Abbott Laboratories. MO-H has received research funding from Abbott Immunology.

## Authors’ contributions

MWB conceived the studies, participated in the design and coordination of the execution of the studies, interpretation of data, and assisted in preparation of the manuscript. GEB performed the statistical analyses and assisted in preparation of the manuscript. DLA, MO-H, BK, and VT coordinated acquisition and interpretation of data. NDM implemented acquisition of data and interpretation of data and LEM implemented acquisition of data, interpretation of data, and assisted in preparation of the manuscript. All authors read and approved the final manuscript.

## Pre-publication history

The pre-publication history for this paper can be accessed here:

http://www.biomedcentral.com/1471-2431/14/136/prepub
